# Pieces of evidences of reliability of the Brazilian version of the Child Executive Functions Battery (CEF-B)

**DOI:** 10.1186/s41155-021-00171-2

**Published:** 2021-02-12

**Authors:** Amanda Guerra, Izabel Hazin, Jean-Luc Roulin, Didier Le Gall, Arnaud Roy

**Affiliations:** 1 Programa de Pós-Graduação de Psicologia, Universidade Federal do Rio Grande do Norte and Laboratoire de psychologie des Pays de la Loire (EA4638), Université d’Angers, 11, boulevard Lavoisier, 49045 Angers, France; 2 Departamento de Psicologia, Universidade Federal do Rio Grande do Norte, Angers, France; 3 Laboratoire de Psychologie et NeuroCognition (UMR 5105), Université de Savoie Mont Blanc, Chambéry, France; 4 Laboratoire de psychologie des Pays de la Loire (EA4638), Université d’Angers, Angers, France; 5 Centre Référent des Troubles d’Apprentissage, Centre de Compétence Nantais de Neurofibromatose, Hôpital Femme-Enfant-Adolescent, CHU de Nantes, Nantes, France

**Keywords:** Executive functions, Child, Neuropsychological assessment

## Abstract

Executive dysfunctions are central symptoms in different neurological, developmental, and context-related conditions. The assessment of these functions is then essential in neuropsychological pediatric clinical practice. Given the need for reliable and valid evaluation batteries for clinical practice in Brazil, this study aimed to present the pieces of evidences of reliability of the Child Executive Functions Battery (CEF-B). A total of 230 Brazilian children with typical development aged between 7 and 12 years participated in the study. Internal consistency was determined by the split-half method, Cronbach’s α, and Ω. In addition, measurements of test-retest reliability and intraclass coefficient were also performed. Retest indicators were mostly weak and moderate (between .43 and .75). Overvall, coefficients show a satisfactory internal consistency reliability for planning and inhibition measures (between .72 and .92). Considering the measures of WM, results were also satisfactory for both α and Ω indexes. This study revealed that the CEF-B has satisfactory internal consistency reliability coefficients. However, several tests have shown low reliability assessed through the test-retest method. In general, findings reveal interesting pieces of initial evidence of reliability of the Brazilian version. The methodological approach could be improved in future studies by including children with executive disorders.

## Introduction

The scientific advancement of child neuropsychology allowed the identification of numerous contexts of brain vulnerability that represent risks of cognitive and behavioral disorders during childhood. Acquired brain injuries, congenital, neurodevelopmental, and neuropsychiatric disorders constitute clinical conditions with potential risk for early neuropsychological dysfunctions. In addition, social and cultural conditions such as maternal nutrition, abuse of alcohol, and other drugs by the mother during pregnancy, inadequate living conditions, physical violence, and sexual abuse, among others, represent potential risk conditions for developmental dysfunctions. These clinical settings greatly impact executive functions (EF) as central symptoms (Craig et al. 2016; Evinç et al. 2018; Lonergan et al. 2019; Mauger et al. 2018; Zelazo 2020). In fact, the early and prolonged physiological maturation of the prefrontal circuits involved in the development of EF imposes a substantial vulnerability to these high-level skills (Dennis 2006). Since these functions are essential to behavioral control and regulation skills, their efficient functioning provides a fundamental basis for psychological development, including cognition, emotions, and social interactions (Diamond 2013). Thus, the identification of early changes on EF in the pediatric population constitutes a substantial clinical and scientific issue.

### Assessment of EF in children

Performance-based tests are still the most usual method for the assessment of EF in children. They provide a standardized and structured evaluation framework that is relatively objective and easy to operate. Over the last 20 years, numerous performance-based tests have been developed or adapted around the world. However, the influence of historical, social, and cultural factors on the emergence of EF in children demands particular considerations when using tests. In Brazil, it is particularly important to consider these aspects because it is a country with a remarkable cultural variability and socioeconomic inequality (Piccolo et al. 2016).

A recent systematic review identified 37 executive measures used in Brazil in the pediatric context (Guerra et al. 2020a). Despite the great variety of tests found, only 13 are allowed to be used in clinical practice by the Federal Council of Psychology. In addition, only eight correspond to measures specially designed for the assessment of EF in children. The remaining five correspond to tests created for the assessment of EF in adults that had their sample expanded for the evaluation in children, without considering the dynamic aspects of executive development. Also, researches that considered at least the three basic executive components are scarce and, to date, no specific battery for assessing EF in Brazilian children is available (Guerra et al. 2020a).

Measurement errors in executive tasks represent another concern that is still often neglected in child evaluation (Van der Linden et al. 2000). The inevitable participation of more basic skills in executive tasks necessarily makes them impure and requires (i) the use of dissociate methods to differ basic skills from executive ones and (ii) to ensure the executive nature of the difficulties encountered by confronting various tasks requiring different non-executive processes (Denckla 1996). This inherent “noise” in executive measures is enhanced in child assessment since the non-executive processes are potentially under development and contribute to age-related variations (Roy et al. 2017). These variables highlight the importance of using appropriate and reliable executive measures for children to meet the objectives of neuropsychological assessment and reduce the potential risk of false positives and false negatives in clinical practice (Guerra et al. 2020a).

### Child Executive Functions Battery

In order to overcome the aforementioned assessment challenges, the Child Executive Functions Battery (CEF-B) was created in France to overcome the scarcity of instruments adapted for the pediatric population (Roy et al. 2020). The CEF-B consists of a set of 12 performance-based tests aimed at children and adolescents between 6 and 16 years old. The battery is based on a child-centered theoretical model and assesses the main executive processes: inhibition, flexibility, working memory (WM), and planning (Diamond 2013). It comprises new experimental tasks and tests that already exist in the international literature but have been modified or expanded to better attend to the pediatric population. Each component (inhibition, WM, flexibility, and planning) is represented by three tests, which are assumed to preferably capture the corresponding dimension. However, this task affiliation is not exclusive because of the interdependent character of EF. One verbal test is proposed per component, while the others are predominantly nonverbal (and mixed, in the case of WM). This approach was conceived in order to cross-reference indicators and provide clinicians with appropriate tests in the case of communication, visuospatial, or gestural disorder (Roy 2015).

The design of a battery specially conceived for children aroused the interest to develop a larger cross-cultural project. Thus, a dynamic intercultural approach has been consolidated with several countries, including Brazil. Given the lack of EF test batteries based on child-centered theoretical models, the CEF-B was adapted to the Brazilian context (Guerra et al. 2020b). However, a test’s adaptation is only the first step to its implementation into a new culture (Borsa et al. 2012). A crucial point regarding the scientific approach of neuropsychological measures is associated with psychometric validity and reliability. These characteristics refer to the legitimacy of the interpretations provided by the test result and the empirical evidence regarding the correspondence between theoretical expectations and the measurement itself (Muniz 2004).

Regarding the CEF-B, preliminary evidence of validity of the French version has been published for studies with children with typical development (Roy et al. 2018), and with different clinical conditions, such as neurofibromatosis type 1—NF1 (Remigereau et al. 2018; Roy et al. 2010, 2014), frontal epilepsy (Charbonnier et al. 2011), and brain tumors (Roche et al. 2018). These initial data indicate a good sensitivity of the battery for the evaluation of EF in pediatric populations. Developmental validity evidences (age-related performance improvement) were found for inhibition tests (Stroop test; *F*(5, 108) = 10.42, *p* < .001). In addition, a good clinical sensitivity was observed through significant statistical differences between clinical and control groups for planning tasks (Rey Osterrieth Complex Figure; *F* (1, 69) = 6.889, *p* = .011—for the NF1 group and *Z* score = 2.89 for frontal epilepsy case) and for flexibility tests (Kids Card Sorting Test; *p* = <.001 for the NF1 group).

Concerning the Brazilian version, a significant improvement with age (developmental validity) was observed between 7- and 12-year-old children (Guerra et al. 2021). A 4-factor EF structure was also found through an exploratory factorial and correlation analysis that corroborate with the theoretical assumption considered in the CEF-B. The same study showed a sensitivity of CEF-B to identify the negative impact of low socioeconomic status on executive development, which agrees with the current literature (Farah 2017; Merz et al. 2019). This study on the trajectory and structure of the EF in the pediatric population of northeast Brazil presents initial evidence of validity which endorses the theoretical and methodological premises of the CEF-B (Guerra et al. 2021). In addition, convergent and divergent validity analyses were also carried out. The findings indicate correct external validity in relation to the three renowned executive tasks used, and good divergent validity compared to the non-executive measures (Guerra 2020). However, given the relevance of providing numerous indicators that attest the importance and utility of a test, we propose to evaluate complementary and different pieces of evidences of reliability of the CEF-B in Brazil.

## Method

### Participants

A total of 230 Brazilian children with typical development aged between 7 and 12 years participated in the study. The sample was homogeneously distributed by age, gender, and type of school (Table 1). Participants were selected based on the following inclusion criteria: (a) signing of the informed consent form by parents or legal guardians; (b) regular registration in public or private school; (c) absence of a history of developmental, neurological, or psychiatric disorders; (d) absence of uncorrected sensory alterations; and (e) scaled score equal or higher than seven points in the WISC-IV Matrix Reasoning and Vocabulary sub-tests.

**Table 1:** Sociodemographic data

	Descriptive
***N***	230
**Age mean (SD)**	9.95 (1.65)
**Age range**	7–12
**Gender (%)**	
*Girls*	116 (50.4)
*Boys*	114 (49.6)
**Type of school (%)**	
*Public*	116 (50.4)
*Private*	114 (49.6)

Note. *SD* standard deviation, % percentages (frequencies)

### Materials

Table 2 presents a brief description of the 12 tests that compose the CEF-B (for a more detailed description of the tasks and variables used, see Guerra et al. 2021). The order of application of the tests that integrate the protocol was defined in a systematic and pseudo-random manner, alternating the investigated executive skills and their verbal/non-verbal nature. In order to limit measurement errors, the variables of the CEF-B were designed to modulate the executive load involved in some multi-composite tests. This approach consists in providing “control” conditions which are supposed to be less demanding on executive processes (i.e., subtracting the Trail A score from Trail B score to “isolate” the contribution of executive abilities in the Trail Making Test; Arbuthnott and Frank 2000).

**Table 2:** Description of CEF-B tests and scientific rationale for controlling measurement errors and methodological bias

	Tests	Description/objective	Proposals for measurement errors and methodological biases control
Inhibition	Stroop	Consists of ignoring the reading of colored words written with non-congruent printing ink (for example, “blue” written in red), to focus on the color of the ink (interfering condition)	-Preliminary control conditions (naming and reading) -Unlimited time, no mistake correction, consideration of time, and errors
	Tapping	Tap or not on the table depending on what the examiner is doing: (1) Go/no go: respond if the examiner types once and inhibit if he types twice. (2) Conflict: antagonistic conditioning (tap once if the examiner taps twice and vice versa) while incorporating a new No go condition (do not tap if the examiner taps with two fingers)	-Preliminary phase of simple conditioning (repeat a motor action in echo)
	Cross-out Joe	Identify and cross out a visual target (Joe) among several morphologically similar distractors	-Evaluate inhibition in a long-term task
Working memory	Verbal updating	Sequentially recall the most recent elements (the last three or four) of a series of letters of varying length	-Task adjusted to span capacities. -Variation in the amount of information to be updated to control the executive load (contrasted with items where no update is required)
	Visuospatial updating	Sequentially recall the most recent items (the last three or four) touched in a series of blocks of varying length	
	Dual task	Simultaneously perform a figure span task and a visuomotor clown head crossing test	- Preliminary execution of both tasks individually - Task adjusted to span capacities
Flexibility	Trail Making Test (TMT)	Connect circles on a sheet of paper that contain numbers or letters, alternating numeric and alphabetical order (1-A-2-B...).	- Control of numerical and alphabetical chain mastery, visual exploration and perceptual-motor skills in two preliminary parts (numbers then letters, respectively)
	Kids Cards Sorting Test (KCST)	Initiate, maintain and change the ranking rule of a series of 48 test cards according to 4 target cards that vary in three dimensions (form, color, number), based on the examiner's feedback	-Only cards that are unambiguous regarding the pairing with the target cards are used - The rules are presented to the child, which reduces the possibility of not understanding the categories
	Frog test	The child must deduce the logical rules according to which a frog moves around several water lilies disposed in a lake. The child must also adapt to the actions of the frog, which changes the movement rule without previous warning.	- Random and variable rule change to make the test less predictable
Planning	Scripts	The child must put in order a sequence of phrases, elaborating a coherent script according to a given title and disconsidering those that are not relevant (intruders)	-New task created to evaluate the child’s ability to anticipate the order necessary for the execution of a daily action
	Rey Osterrieth Complex Figure (ROCF)	Copy the ROCF spontaneously and progressively recopy the figure according to a program consisting of five successive stages of different colors.	-Measurement of the facilitating effect of copying with the program in contrast to spontaneous copying -Rigorous and objective instructions for the evaluation of the precision and location of the figure elements
	8 Mazes	The test comprises eight mazes of increasing difficulty. For each maze, a dinosaur has to find its way out. The test requires the child to draw, with a pencil, the path connecting the starting point to the maze’s exit.	-Consider time and error

### Procedure

The study was conducted in 14 public and private schools in Natal, Parnamirim, and Elói de Souza in the Rio Grande do Norte state. The project was submitted to and approved by the Research Ethics Committee of the Federal University of Rio Grande do Norte, under code 48383715.1.0000.5537. After the informed consent term was signed by legal guardians, children were evaluated using the vocabulary and matrix reasoning subtests in a single session lasting approximately 20 min at the school itself and during the regular school term.

The selection of participants was carried out in collaboration with the coordinators and teachers of each institution, who indicated the children to participate in the study. Coordinators and teachers were asked to indicate children with no suspicion or diagnosis of neurodevelopment disorders. We randomly selected the participants from the list of children indicated by school professionals which were also authorized by parents to participate in the survey. It should be noted that a questionnaire was completed by the parents when signing the consent form to ensure that the children who participated in the research did not present a history of developmental, neurological, or psychiatric disorders. A total of 264 signed consent forms were collected, and 244 children and adolescents were submitted to the WISC-IV subtests vocabulary and matrix reasoning. Fourteen of the participants presented weighted points below seven in one of the subtests and were therefore excluded from the sample because they did not meet one of the inclusion criteria.

All participants were individually evaluated in a quiet room in their school or home environment. The tests were administered by trained neuropsychologists using standardized instructions. The assessment of the children consisted of the application of the entire CEF-B, requiring two or three assessment sessions with a duration of approximately 30–40 min each, depending on the age of the child. The tests were systematically presented in the same order. The first session included: 8 Mazes, Stroop, Visuospatial updating, Scripts, and Tapping tasks. The second session contemplated the Rey Complex Figure, Trail Making Test, Dual task, Kid Card Sorting test, Cross-out Joe, Verbal updating test, and Frog test. In case an additional session was needed, four tests were presented per session in the aforementioned order.

The second phase comprised the application of the tests that were selected for the retest method. This step was carried out 4 to 6 weeks after the last assessment session of the child. One 40-min session was required to perform the 6 CEF-B tasks that were retested, which were administered by the same evaluator in the following order Stroop, Tapping, Kids Cards Sorting Test, TMT, Dual task, and Frog test.

### Statistical analyses

The reliability of the CEF-B was verified by several methods. In fact, the study of the reliability of EF measurements is complex because analysis by internal consistency and split-half methods are in most cases not applicable. In addition, test-retests can affect the validity of the second measurement, since time measures can be associated with learning effects between the two sessions (Soveri et al. 2018). Finally, the examiner is sometimes an important source of measurement error, which means that reliability among examiners must also be calculated (Urbina 2007). For this reason, different indicators were used for the tasks that compose the CEF-B.

Retest was applied for tasks in which the time factor was central to the accomplishment of the task and when the use of another method was not applicable. It was applied for all flexibility measures, two inhibition measures (Stroop and Tapping) and one WM test (Dual task). For tests in which it was possible to use different methods of reliability other than retest, we prioritized the use of classical methods such as split-half (Spearman-Brown formula is used to correct the effect of splitting the number of items), internal consistency, and intraclass coefficients. In the cases of the Scripts, the 8 Mazes, and for Verbal and visuo-spatial updating tasks, two indicators of internal consistency (Cronbach’s alpha and omega—Cronbach 1951; McDonald 1985, 1999) were applied instead. Also, the split-half method (even and odd items) was used for the 8 Mazes test. Pearson’s correlation was calculated for both parts of the Cross-out Joe test. Since part B of this test corresponds to the mirrored version of part A, the purpose of this measure was to demonstrate the equivalence of these two steps. To this end, we calculated the correlation between A-B (A being applied first, followed by B), and we calculated the correlation between parts when they were applied in the opposite order (B-A). Regarding Rey’s figure, an intraclass coefficient was calculated for three indices: the copy score, the program score, and the planning index. For the calculation of this coefficient, four different examiners corrected the figures. All statistical analyses were performed using the “Psych” package (Revelle 2020) of the R software (R Core Team 2020). For all analyses, the significance level was set at .05.

## Results

### Test-retest reliability

Table 3 summarizes, by domain and task, the reliability index obtained with the retest method. The coefficients observed are mostly low, but some are moderate. In fact, coefficients of reliability vary according to the type of measurement, for example for Tapping (Tapping *Go/No-Go Time*—*r* = .18; while the others task indicators vary between .43 and .44), Dual task (Dual task *Evolution clowns*—*r* = .23, while the *Score Mu* and *Evolution digits* vary from .56 and .57), and Frog test (Time = .43 and Score = .70). However, for other tests such as KCST (time = .59, categories = .60, perseveration = .75) and Stroop (Time = .53; Error = .50), the coefficients seem to be more homogeneous. Significant practice effects were found for all variables of two (KCST and Frog test) of the six tasks assessed by the retest method. Overall, this result revealed an improvement trend in children’s performance at the retest phase (Table 3).

**Table 3:** Reliability coefficients obtained through the test-retest method

EF	Test	Variable	*N*	*Test*		*Retest*		*t*	*r*_*xx*_
				*M*	*SD*	*M*	*SD*		
**Inhibition**	**Stroop**	*Time*	28	92.9	54.3	67.7	55.7	1.94	.53
		*Error*		3.1	3.7	1.7	2.9	1.86	.50
	**Tapping**	*Go/No-Go Time*	31	6.7	11.3	3.1	8.1	1.54	.18
		*Go/No-Go Error*		1.3	2.0	0.8	1.1	1.30	.43
		*Conflict Time*		22.2	13.4	24.2	30.2	− .02	.44
		*Conflict Error*		2.4	2.8	1.9	2.0	1.31	.43
**Working memory**	**Dual task**	*Evolution digit span*	31	101.1	54.4	99.2	36.3	.58	.57
		*Evolution clowns*		96.1	13.2	102.1	13.8	1.73	.23
		*Mu Score*		98.6	30.2	100.7	19	− .04	.56
**Flexibility**	**TMT**	*Flexibility index*	28	38.4	41.9	41.4	54	1.42	.27
	**KCST**	*Time*	30	227.9	68,2	214,8	75.9	.76	.60
		*Categories*		2.9	1.2	3.6	1.6	− 2.37	.59
		*Perseverations*		7.5	4.8	5.5	4.3	2.71	.75
	**Frog test**	*Time*	30	262.8	88	216	63.7	2.97	.43
		*Score*		52.6	9.8	55.7	9.7	− 2.53	.70

Note. *M* Mean, *SD* Standard deviation, *rxx* reliability coefficient

### Split-half method, internal consistency, and intraclass coefficients

The results obtained through the split-half method, internal consistency, and intraclass coefficients are described in Table 4. Coefficients show overall satisfactory reliability for planning and inhibition measures (between .72 and .92). Considering the measures of WM, results were also satisfactory for both alpha and omega indexes.

**Table 4:** Reliability indicators using the split-half method, internal consistency, and intraclass coefficients for planning, WM, and inhibition tests

EF	Test	Variables	N	*rxx*	alpha	*ωt*	ICC
**Planning**	Scripts	*Time*	171	–	.86	.86	–
	8 Mazes	*Time*	221	.76	.83	.83	–
		*Errors*	221	.86	.86	.87	–
	ROCF	*Spontaneous copy*	207	–	–	–	.92
		*Copy with program*	207	–	–	–	.86
		*Planning index*	207	–	–	–	.89
**Working Memory**	Updating tasks	*Verbal updating*	150	–	.82	.84	–
		*Visuospatial updating*	195	–	.85	.86	–
**Inhibition**	Cross-out Joe	*Correlation A-B*	184	.77	–	–	–
		*Correlation B-A*	29	.71	–	–	–

Note. *ωt* omega total, *rxx* reliability coefficient, *alpha* cronbach's alpha, *ROCF* Rey-Osterrieth Complex Figure, *ICC* intra-class coefficients

## Discussion

The aim of the present study was to present the additional psychometric properties of the CEF-B in a sample of 7–12-year-old children from Northeastern Brazil. Results revealed initial adequate pieces of evidences of reliability for the CEF-B for the assessment of EF in Brazilian children.

Different alternatives are reported in the literature to evaluate reliability (Gregory 2010). It is currently suggested to use several methods that provide pieces of evidences of the test’s reliability, similar to what is observed for validity. In fact, it should be noted that reliability is a characteristic of the test scores and not the test itself. In this sense, these various methods and the choice of variables produce complementary but sometimes contradictory estimates. In executive tasks, the study of the reliability of measurements is complex because test-retests can affect the validity of the second measurement (Soveri et al. 2018). Also, analysis by internal consistency and split-half methods are in most cases not applicable. Finally, the examiner is sometimes an important source of measurement error, which means that reliability among examiners must also be calculated (Urbina 2007).

To examine the pieces of evidences of reliability in the Brazilian CEF-B version, we used different methods according to the nature of each executive test. For tasks where the time factor was central to the accomplishment of the task (half of the tasks), the retest was applied. In general, retest indicators were mostly weak and moderate. These results indicate a fluctuating retest stability depending on the measurement used. Such variability can be explained either by the different strategies used by the child in test and retest situations or by learning effects. The lowest values concern the Tapping task (*r* = .18), for which the results seem to be very dependent on testing conditions. In fact, this finding could be related to examiner bias, since the delay between the presentation of the stimulus and the children’s response can vary between examiners. This task would be more susceptible to this type of variation given the need to react according to the child's behavior. The reaction time of the child may condition the time of presentation of the stimulus by the examiner. A solution for this issue could be the use of a pre-defined delay, which could be achieved by converting the test to a digital version.

Another task that also seems to have problematic evidence concerns the Dual task (*Evolution clowns* variable; *r*=.23). Results observed on the retest may reflect changes in strategies (prioritizing the motor task over the cognitive task and vice versa) that lead to indirect effects on reliability scores. The same reasoning is valid for the TMT index. It is important to note that this variation has also been observed in previous studies on the assessment of EF. In fact, when several EF tasks are administrated, performance in these tasks is often poorly correlated and reliability rates are low (e.g., Lemay et al. 2004; Soveri et al. 2018; Willoughby et al. 2017). This weak reliability, most often associated with the test-retest situation, is usually explained by the fact that these EF tasks are susceptible to a practical effect that partially distorts this assessment.

For tests in which it was possible to use different methods of reliability other than test-retest, we prioritized the use of classical methods such as split-half, internal consistency, and intraclass coefficients. Regarding the reliability assessed through Cronbach’s alpha and omega coefficients, satisfactory indicators were found for Scripts, 8 Mazes, and the updating tasks (between .82 and .87). According to the Brazilian Federal Council of Psychology (Conselho Federal de Psicologia – CFP 2003; Primi et al. 2004), the minimal acceptable value for these indices is .60. In addition, some authors have suggested the following value classification: .80–.90, very good; .70–.80, respectable; .65–.70, acceptable; .60–.65 undesirable; and below .60, unacceptable (Freire and Almeida 2001). It is also important to note that the split-half method indicators for the 8 Mazes tests were also high, showing a good internal consistency of the task. The coefficients for Cross-out Joe were also acceptable, both under the A-B application order (*r*=.77), as well as under the reverse order (B-A; *r*=.71). This result also indicates an adequate internal consistency of the task.

Regarding the ROCF, reliability issues are mostly associated with the method of task correction. In fact, the correction of the task is often considered as subjective. In the CEF-B version of the test, we adopted more rigorous and objective instructions for the evaluation of the precision and location of the figure elements drawn by the children. Thus, the agreement between examiners was measured by the intraclass coefficient for the three measures of the test. Results showed a good consensus (.86 to .92) and indicate that a more rigorous and objective correction of the test may lead to more stable scores between examiners.

It should be mentioned that in addition to the findings regarding reliability evidences, the study by Guerra et al. (2021) allowed to expand the CEF-B adaptation process (Guerra et al. 2020b) by presenting preliminary normative data and evidences of validity that favor the use of this version in the Brazilian context. Results revealed evidences of developmental validity and a factorial structure compatible with the theoretical proposition of the battery, revealing consistent evidence of construct validity. In addition, data regarding the negative impact of low SES on EF performances also corroborate with the literature and show a satisfactory sensitivity of the battery in identifying these potential differences (Guerra et al. 2021).

Although classical measures of validity and reliability are necessary to prove the scientific nature of the battery, only the proof of clinical utility regarding the dissociation of a deficit and the expected result is able to truly attest its relevance. Thus, one of the future objectives of the CEF-B project in Brazil is to provide clinical data in order to assess the clinical sensibility of the battery. This endeavor is currently being carried out in several research centers in Rio Grande do Norte.

The main limitation of the present study concerns the sample size and its generalization to the Brazilian context. In fact, Brazil’s population and its social and economic diversity require a wider sample in order to assure the representativeness of its cultural diversity (see Guerra et al. 2020a for a review). In particular, the sample used for the test-rest reliability should be extended in future studies. In addition, the lack of data from children with clinical conditions in the sample, which would lead to a higher variance in data, constitute important limitations to the potential for generalization of the obtained results.

## Conclusions

This study revealed that the tests of the CEF-B presented satisfactory pieces of evidence of reliability assessed through split-half method, internal consistency, and intraclass coefficients. On the other hand, several tests have shown low reliability retrieved by the test-retest method. Although these findings reveal interesting pieces of initial evidence of reliability of the Brazilian version, the methodological approach could be refined in future studies in order to include children with executive disorders and to provide the assessment of the clinical sensitivity of the CEF-B.

## Data Availability

Data sharing is not applicable to this article as no datasets were generated or analyzed during the current study.
